# Cooperative Effect of miR-141-3p and miR-145-5p in the Regulation of Targets in Clear Cell Renal Cell Carcinoma

**DOI:** 10.1371/journal.pone.0157801

**Published:** 2016-06-23

**Authors:** Julia Liep, Ergin Kilic, Hellmuth A. Meyer, Jonas Busch, Klaus Jung, Anja Rabien

**Affiliations:** 1 Department of Urology, Charité - Universitätsmedizin Berlin, Berlin, Germany; 2 Berlin Institute for Urologic Research, Berlin, Germany; 3 Institute of Pathology, Charité - Universitätsmedizin Berlin, Berlin, Germany; Sapporo Medical University, JAPAN

## Abstract

**Background:**

Due to the poor prognosis for advanced renal cell carcinoma (RCC), there is an urgent need for new therapeutic targets and for prognostic markers to identify high risk tumors. MicroRNAs (miRNAs) are frequently dysregulated in tumors, play a crucial role during carcinogenesis and therefore might be promising new biomarkers. In previous studies, we identified miR-141-3p and miR-145-5p to be downregulated in clear cell RCC (ccRCC). Our objective was to investigate the functional association of these miRNAs, focusing on the cooperative regulation of new specific targets and their role in ccRCC progression.

**Methods:**

The effect of miR-141-3p and miR-145-5p on cell migration was examined by overexpression in 786-O cells. New targets of both miRNAs were identified by miRWalk, validated in 786-O and ACHN cells and additionally characterized in ccRCC tissue on mRNA and protein level.

**Results:**

In functional analysis, a tumor suppressive effect of miR-141-3p and miR-145-5p by decreasing migration and invasion of RCC cells could be shown. Furthermore, co-overexpression of the miRNAs seemed to result in an increased inhibition of cell migration. Both miRNAs were recognized as post-transcriptional regulators of the targets EAPP, HS6ST2, LOX, TGFB2 and VRK2. Additionally, they showed a cooperative effect again as demonstrated by a significantly increased inhibition of HS6ST2 and LOX expression after simultaneous overexpression of both miRNAs. In ccRCC tissue, LOX mRNA expression was strongly increased compared to normal tissue, allowing also to distinguish between non-metastatic and already metastasized primary tumors. Finally, in subsequent tissue microarray analysis LOX protein expression showed a prognostic relevance for the overall survival of ccRCC patients.

**Conclusion:**

These results illustrate a jointly strengthening effect of the dysregulated miR-141-3p and miR-145-5p in various tumor associated processes. Focusing on the cooperative effect of miRNAs provides new opportunities for the development of therapeutic strategies and offers novel prognostic and diagnostic capabilities.

## Introduction

Renal cell carcinoma (RCC) is the most common kidney tumor in adults, representing approximately 3% of all malignant tumors in humans [1]. The RCCs comprise a very heterogeneous group of tumors. The most common histological subtype is the clear cell renal cell carcinoma (ccRCC) which represents about 70% of all RCC [2]. In general, primary RCC shows no early clinical symptoms and is most often first discovered by routine investigations. Approximately 25% of diagnosed RCCs already present metastasis and another 20–30% develop metastasis following surgery [2]. Unfortunately, there is a very poor clinical outcome for patients with metastatic RCC of just 1–2 years overall survival after nephrectomy [3]. Although there is a number of therapeutic options like tyrosine kinase inhibitors and angiogenesis inhibitors [2], only an unsatisfactory response rate can be achieved so far. In addition to the side effects of systemic therapy, drug resistance of tumors is a major problem that needs to be solved [4]. There is a well-founded hope to use a better insight into molecular patterns in order to ensure an adequate risk stratification. This will identify tumors with an increased risk for the transition from a local disease to a tumor generating distant metastasis. In addition, there is still an urgent need for new molecular targets to develop specific agents for targeted therapy with fewer side effects.

MicroRNAs (miRNA) are small, non-protein coding molecules which play a key role in the regulation of gene expression by post-translational inhibition of their specific target transcripts. They are found to be involved in the control of several tumor associated processes like cell proliferation, apoptosis, migration and invasion and to be frequently dysregulated in tumor tissue [5,6]. MiRNAs are extensively studied and discussed as a new class of promising biomarkers for the diagnosis and prognosis of tumors. For ccRCC we could already establish a comprehensive miRNA expression profile, in which predominantly downregulated miRNAs were identified, like miR-141-3p and miR-145-5p [7]. Reduced expression of these both miRNAs in ccRCCs was also confirmed by later studies [8–12]. As we have shown in our previous study that the observed inhibition of miR-141-3p and miR-145-5p in RCC cells is mediated by epigenetic mechanisms [7], we focused on the functional role of these miRNAs in RCC, here.

Both, miR-141-3p and miR-145-5p are described to be dysregulated and to act as tumor suppressors in various tumor entities. MiR-141-3p is a member of the miR-200 family, which is found to be generally involved in tumor progression and especially in the epithelial-mesenchymal transition (EMT) of many tumor entities [13]. Thus, the miR-200 family is mainly involved in processes of migration and invasion. The main tumor suppressive function of miR-145-5p seems to be promotion of apoptosis [14], but it is also involved in further processes concerning tumor progression and metastasis formation [15].

The tumor suppressive activity of miRNAs is determined by their specific targets. However, there is a complex network of miRNA and target interactions, so that one single miRNA can affect several hundred different targets, and on the other hand one target can be regulated by various miRNAs. To investigate the role of miRNAs in tumorigenesis, their individual major targets have to be identified in order to decipher a part of this complex network. There are several algorithms available for the prediction of putative targets, which perform target search based on sequence complementarity as well as on sequence conservation and steric and pairing stability [16]. But since the required sensitivity and specificity of these models is too low, a confirmation by experimental analysis is essential. In most cases, the effect of one single miRNA toward the expression of its target is just slight. However, many miRNAs can inhibit multiple targets of one pathway, whereby this effect can be enhanced. Combining miRNAs with the same targets may result in a cooperative effect and therefore in an effective inhibition or stimulation of appropriate targets or mechanisms [17]. It has been described that the regulatory effect of multiple miRNAs on one common target can be significantly enhanced by a combined inhibition [18]. This could also be a new strategy to improve current therapeutic approaches of miRNA-based therapies for RCCs.

The aim of our study was to identify new targets regulated by both miR-141-3p and miR-145-5p in ccRCC, in order to obtain a deeper insight into the network and to better understand the role of these miRNAs and their targets in carcinogenesis of ccRCC.

## Materials and Methods

### Patients and tissue samples

Malignant and adjacent non-malignant tissue was obtained from patients with ccRCC during nephrectomy at the Department of Urology, University Hospital Charité in Berlin. All patients provided written informed consent for the use of their tissue in research. The study was approved by the ethics committee (EA1/153/07 and EA1/153/12: ‘microRNAs as diagnostic and prognostic signatures in urological tumors’) and conducted in compliance with the Declaration of Helsinki. The tumor samples were classified according to the 2002 TNM classification and the Fuhrman grading system [19,20]. After surgical resection, all tissue samples for RNA expression analysis were frozen in liquid nitrogen and stored at -80°C until RNA extraction. Tissue for immunohistochemical analysis was formalin-fixed and paraffin-embedded (FFPE).

For quantitative real-time reverse-transcription PCR (RT-qPCR) analysis we used tissue from malignant tumors and the adjacent non-malignant kidney from 27 patients with ccRCC collected between 2005 and 2012.

For immunohistochemical analysis of protein expression in RCC, a tissue microarray (TMA) with FFPE tissue, including 322 ccRCC patients nephrectomized between 1993 and 2004 was used [21]. Clinicopathological characteristics for all patients are listed in Table 1.

**Table 1:** Clinicopathological patient characteristics.

		ccRCC patients (n = 27)	ccRCC patients (n = 322)
		**mean (range)**	
Age in years		63 (42–85)	61 (25–84)
Follow-up in months		23 (0–71)	101 (0–186)
		**n (%)**	
Sex	female	8 (30)	115 (36)
	male	19 (70)	207 (34)
pT stage	pT1	11 (41)	193 (60)
	pT2	2 (7)	15 (5)
	pT3	14 (52)	111 (34)
	pT4	0	3 (1)
Fuhrman grade	G1	0	47 (14)
	G2	20 (74)	234 (74)
	G3	7 (26)	38 (11)
	G4	0	3 (1)
Nodal status	N0/Nx	25 (93)	304 (94)
	N1	2 (7)	18 (6)
Metastases	M0/Mx	15 (56)	292 (91)
	M1	12 (44)	30 (9)
Deaths		10 (37)	118 (37)

### RNA extraction and RT-qPCR

Total RNA including miRNAs was isolated from the collected frozen ccRCC tissue and renal cancer cell lines using the miRNeasy Mini Kit (Qiagen, Hilden, Germany) in accordance to the manufacturer’s protocol and as previously described [22]. RNA quantity was determined with a NanoDrop 1000 Spectrophotometer (NanoDrop Technologies, Wilmington, DE, USA) and absorbance ratios of A260/230 and A260/280 were calculated. RNA quality was analyzed by the RNA integrity number (RIN) using a Bioanalyzer 2100 (Agilent Technologies, Santa Clara, CA, USA) with a RNA 600 Nano Lab Chip. For RT-qPCR analyses only samples with RIN above 5 were included.

For mRNA quantification, cDNA synthesis was performed using the Transcriptor First Strand cDNA Synthesis Kit (Roche) and PCR was done on the Light Cycler 480 (Roche) using the QuantiTec SYBR Green PCR Kit or Universal Probe Library (UPL) probes (Roche) with the LightCycler 480 Probes Master (Roche). Briefly, in a total volume of 20 μl 0.5–1 μg total RNA was reverse transcribed. For PCR measurements, 1 μl of cDNA was amplified with 2.5 μM transcript-specific primers (TIB Molbiol, Berlin, Germany) with the respective assay master. The reactions for SYBR Green PCR were performed at 95°C for 15 min, followed by 45 cycles with denaturation at 94°C for 15 s, variable primer annealing temperature for 30 s (S1 Table) and elongation at 72°C for 30 s. For PCR with specific UPL probes (S1 Table), reactions were performed at 95°C for 10 min, followed by 45 cycles with denaturation at 94°C for 10 s, primer annealing at 60°C for 20 s and elongation at 72°C for 1 s.

For mature miRNA quantification, the TaqMan MicroRNA primer assays (Life Technologies GmbH, Applied Biosystems, Darmstadt, Germany) (S2 Table) were used according to the manufacturer’s protocols, the MIQE guidelines [23] and as previously described [22,24]. Briefly, 6.67 ng of total RNA were transcribed into cDNA using TaqMan MicroRNA reverse transcription Kit (Applied Biosystems) and miRNA-specific stem-looped primers in a total volume of 10 μl. The relative quantification was performed on the Light Cycler 480 Instrument (Roche Applied Science, Mannheim, Germany) using 1 μl cDNA, 1x TaqMan Universal PCR Master Mix, No AmpErase UNG and miRNA-specific primers in a total volume of 10 μl in accordance to the manufacturer's recommendations.

The PCR measurements were done in triplicates, including non-template control and interplate controls in each PCR run. PCR data were analyzed by qBase^PLUS^ software (Biogazelle NV, Gent, Belgium). For normalization of the miRNA expression data the reference gene combination miR-28, miR-103 and miR-106a was used [22]. For normalization of the mRNA expression data, the reference gene peptidylproline isomerase A (PPIA) was used [25].

### Cell culture

The human RCC cell lines 786-O and ACHN (American Type Culture Collection, Manassas, VA, USA) were used for transfection experiments. 786-O cells were cultured in RPMI 1640 (Life Technologies GmbH, Invitrogen, Darmstadt, Germany) and ACHN cells were maintained in Eagle's Minimum Essential Medium (Biochrom GmbH, Berlin, Germany) under standard conditions (37°C, 5% CO_2_). All media were supplemented with 10% fetal calf serum (PAA Laboratories, Pasching, Austria) and penicillin/streptomycin (PAA Laboratories). The identity of both cancer cell lines was verified by Multiplexion GmbH (Heidelberg, Germany).

For transfection, cells were seeded into 6-well plates in a final density of 0.8x 10^5^ 786-O cells or 2x 10^5^ ACHN cells per well. The next day, the cells were transfected with pre-miRNA precursors (miR-141, miR-145) or negative control pre-miR-NC#1 (NC#1) (Life Technologies GmbH, Ambion, Darmstadt, Germany) (S3 Table) in a final concentration of 30 nM of each miRNA using Lipofectamine2000 transfection agent (Invitrogen). After 48 h incubation, cells were lysed and total RNA was extracted. Efficiency of transient cell transfection was verified by different control experiments (S1 Fig).

To assess the migratory phenotype of 786-O upon miRNA transfection, BD BioCoat^TM^ Tumor Invasion System (BD Biosciences, Heidelberg, Gremany) was used according to the manufacturer’s recommendations. For this transwell assay we used BD Falcon HTS FluoroBlok 24-multiwell insert plates with a pore size of 8 μm (BD Biosciences). Cells were transfected in 6-well plates as described above. After 24 h incubation, cells were seeded into uncoated (for migration rate) and matrigel-coated (for invasion rate) 24-multiwell insert plates. In the subsequent incubation time of 20 h, cells were able to migrate through the membrane following the serum gradient from medium without serum in the upper chamber into medium with 5% serum in the lower chamber. Quantification of migrated/invasive cells was done by 1 h incubation with 4 μg/μl calcein (Invitrogen) in HBSS (Life Technologies GmbH, Gibco, Darmstadt, Germany) and fluorometric detection of stained cells at wavelengths of 485/538 nm (Ex/Em) on the bottom side of the membrane by using the Fluoroskan Ascent plate reader (Thermo Lab Systems, Langenselbold, Germany).

### In silico prediction of microRNA targets

In silico target search for putative miRNA targets was performed using the miRWalk software (http://www.umm.uni-heidelberg.de/apps/zmf/mirwalk/) [26]. We chose genes containing gene regions with the following search criteria: 2000 upstream flanked + 3`UTR and options: longest transcript; min seed length = 7; p-value ≤ 0.05. For our analysis, we focused on potential targets, which are already described in literature to be involved in cancerogenesis and tumor progression.

### mRNA microarray

For mRNA microarray analysis, data of our previously described and analyzed Human Genome U133 Plus 2.0 Arrays (Affymetrix, Santa Clara, CA, USA) microarray were used [27]. The corresponding data are archived under the GEO Accession No. GSE66272 and GSE66271. As threshold we used p ≤ 0.05 and valid signal in min. 50% of patients.

### Immunohistochemistry

Immunohistochemistry was performed on 4 μm tissue slices of a tissue microarray. The tissue was deparaffinized with xylene, gradually hydrated and antigens were demasked in a pressure cooker in 0.001 M EDTA buffer. Non-specific binding sides were blocked with Protein Block Reagent (Dako Deutschland GmbH, Hamburg, Germany) for 10 min at room temperature (RT). LOX rabbit polyclonal antibody was incubated for 1 h at RT (1:50) (Cat. No. ab31238, Abcam, Cambridge, MA, USA). Afterwards, secondary donkey polyclonal antibody conjugated with alkaline phosphatase (1:500) (Cat. No. 711-056-152, Jackson, ImmunoResearch Laboratories, Inc, West Grove, PA, USA) was applied for 30 min at RT. For visualization, slides were stained with Fast Red (Sigma-Aldrich, Munich, Germany) and nuclei were counterstained with haemalaun (Dr. K. Hollborn & Söhne, Leipzig, Germany). Immunohistochemical analysis was performed on a tissue microarray containing 322 ccRCC tissues, each represented by three spots of 1.0 mm diameter. LOX staining was examined separately for the apical (LOXa) and cytoplasmic (LOXc) localization each scored as negative (0), moderately positive (1) and highly positive (2) staining by an expert uro-pathologist (EK) as an average for the three spots of the same case.

### Data analysis and statistics

For statistical analysis of the data, GraphPad Prism 5.04 (GraphPad Software, San Diego, CA, USA) and SPSS 23 (IBM SPSS Statistics Software, Somers, NY, USA) were used. Calculations were performed by parametric (Student’s t-test) and non-parametric tests for paired (Wilcoxon test) and unpaired (Mann-Whitney test) data. Associations were determined by Spearman’s rank correlation or Chi-square test. Diagnostic impact of parameters was shown in a Receiver Operating Characteristic (ROC) analysis by calculating the area under the curve (AUC) value. Survival analysis were performed by Kaplan-Meier analysis using the log rank test. In all cases p-values < 0.05 were considered as statistically significant.

## Results

### Inhibition of cell migration by miR-141-3p and miR-145-5p in 786-O cells

**Fig 1:**
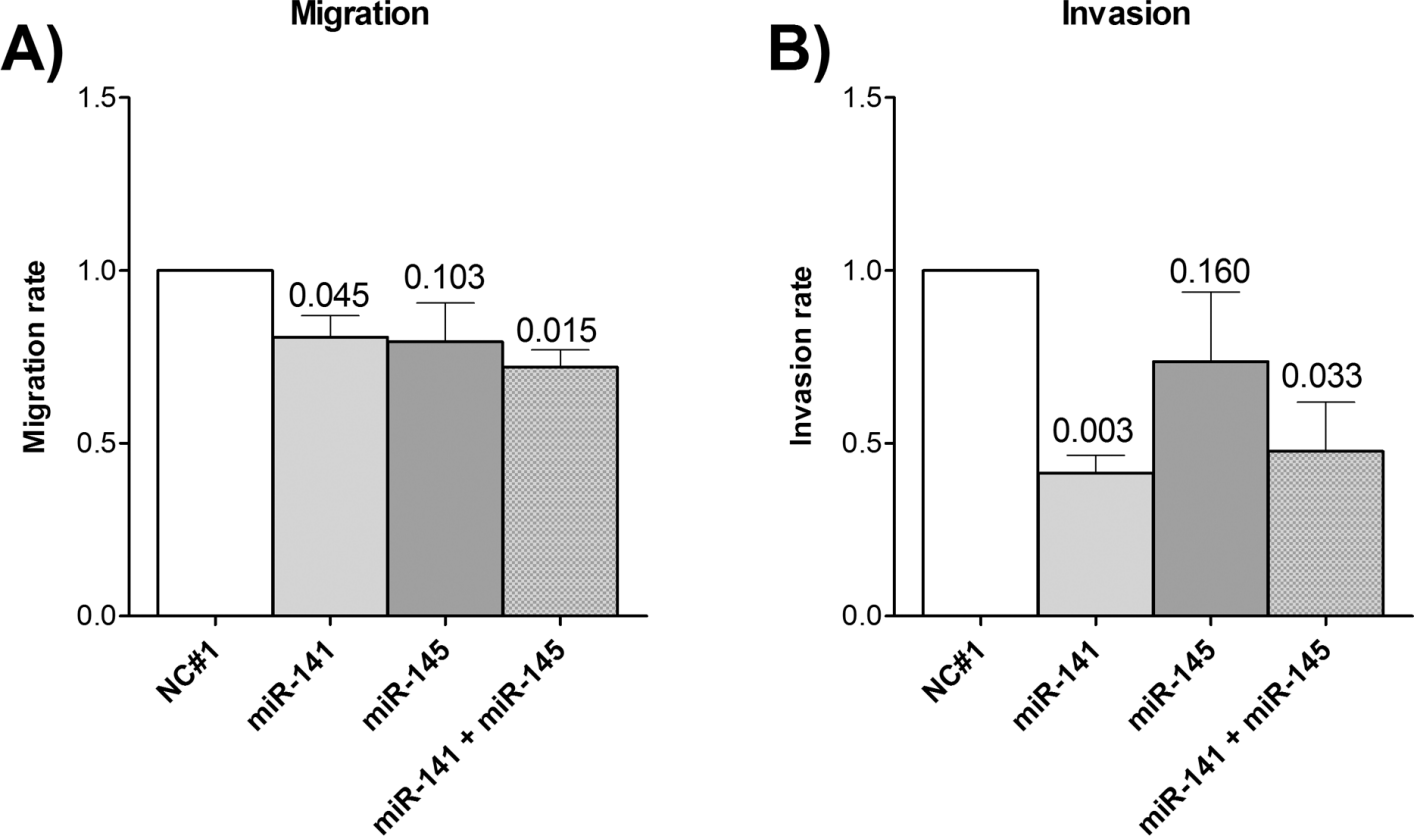
Effect of miRNAs on cell migration and invasion of renal cancer cell line 786-O. A) Cell migration and B) cell invasion of miRNA overexpressing 786-O cells were analyzed by a transwell assay. Migration/invasion rate is given in relation to negative control (mean ± SEM). P-values are given as numbers above bars. NC#1 = negative control; n = 3; t-test (one-tailed).

miR-141-3p and miR-145-5p are described to be negative regulators of cell migration and invasion. To analyze the effect of the miRNAs in the RCC cell line 786-O, we performed a migration and invasion assay. Here, miR-141-3p had a strong inhibitory effect on migration and invasion and also miR-145-5p tended to inhibit both mechanisms (Fig 1). Additionally, the combination of both miRNAs seemed to have a slight cooperative effect on migration compared to the single miRNAs.

### Selection of predicted targets for miR-141-3p and miR-145-5p

miRWalk predicted 1099 putative targets for miR-141-3p and 947 putative targets for miR-145-5p. Since in most cases there was only a slight inhibitory effect of one miRNA, we wanted to identify also targets which might be regulated by both miRNAs. Thus, we compared the two lists and obtained 100 targets, predicted to be targets of miR-141-3p as well as of miR-145-5p (S4 Table). Focusing on potential targets which were already described to be involved in carcinogenesis, we performed a literature search for these 100 common targets. Because of their oncogenic role in tumor-associated pathways, we selected 10 targets, listed in Table 2, for further analysis.

**Table 2:** Predicted cancer targets of miR-141-3p and miR-145-5p by miRWalk.

Target	Character	Reference
EAPP	promoting proliferation	[28]
FRMD4	promoting tumor growth and metastasis	[29]
HS6ST2	pro-migratory	[30]
ITGB3	promoting proliferation and migration	[31]
LOX	pro-migratory	[32]
NRP2	promoting angiogenesis	[33]
SLC16A3	anti-apoptotic	[34,35]
TGFB2	involved in growth factor signaling	[36]
TNFSF4	involved in TNF signaling and tumor immune response	[37]
VRK2	anti-apoptotic	[38]

### miR-141-3p and miR-145-5p regulate expression of various predicted targets in RCC cell lines

**Fig 2:**
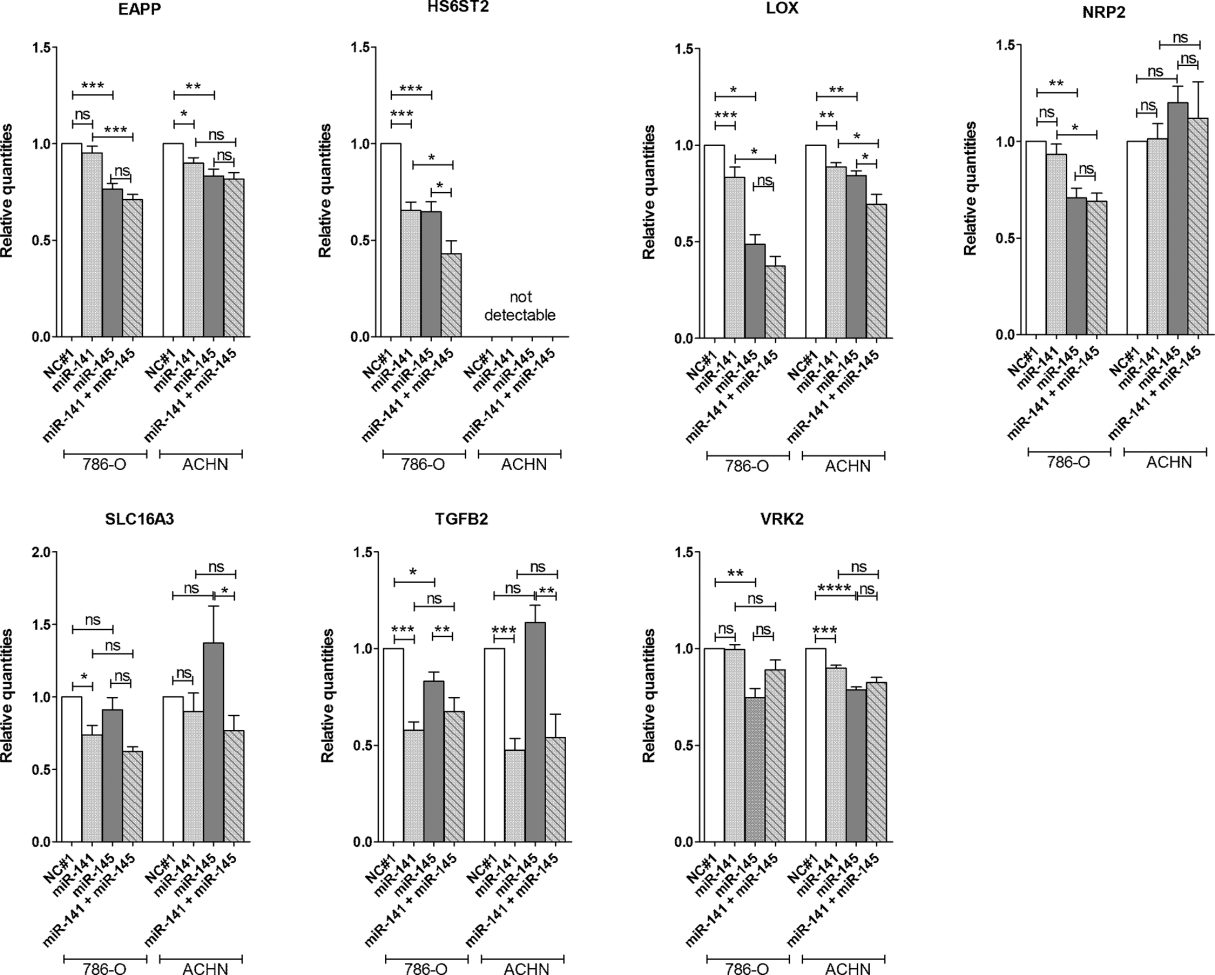
Effect of miRNA overexpression on target expression. MiR-141-3p and/or miR-145-5p were overexpressed in renal cancer cell lines 786-O and ACHN for 48 h. Effect on mRNA expression of putative targets, which were predicted to be regulated by miR-141-3p and miR-145-5p, was determined by RT-qPCR. Data are shown as mean (± SEM) relative to NC#1. NC#1 = negative control, n = 3, student’s t-test (two-tailed); *p < 0.05; **p < 0.01; ***p < 0.001; ****p < 0.0001; ns = not significant.

To analyze if miR-141-3p and miR-145-5p can affect the expression of the predicted targets in renal cancer cells, we overexpressed one or both miRNAs in cell lines 786-O and ACHN. The five targets EAPP, HS6ST2, LOX, TGFB2 and VRK2 were downregulated by each of the miRNAs in at least one of the two cell lines (Fig 2). Furthermore, the target NRP2 was regulated by miR-145-5p only and SLC16A3 was regulated by miR-141-3p only. The other targets showed no effect.

Interestingly, by combined overexpression of miR-141-3p and miR-145-5p we detected a significantly increased reduction of HS6ST2 and LOX expression (Fig 2). HS6ST2 was generally not detectable in the ACHN cell line, but there was a high expression in 786-O cells. This strong HS6ST2 expression in 786-O cells was reduced by 30% after miR-141-3p or miR-145-5p overexpression. Combination of both miRNAs then even reached a decrease in HS6ST2 expression of more than 50%. The miRNA-mediated inhibition of LOX was different in the two cell lines. There was just a slight inhibition by miR-141-3p in both cell lines, but in 786-O cells miR-145-5p reduced LOX expression by about 50% and the combined overexpression of miR-141-3p and miR-145-5p reached an additional decrease of LOX expression of more than 60%.

A strong decrease was also observed for TGFB2 expression after miR-141-3p overexpression. Here, the target expression was reduced by about 50% just by overexpressing one single miRNA.

Because of the interesting cooperative effect of miR-141-3p and miR-145-5p in the regulation of HS6ST2 and LOX, these two targets were selected for further investigations in ccRCC tissue. Both targets contain one or two binding sides for miR-141-3p and miR-145-5p in different regions of the 3’UTR of their transcripts (S2 Fig).

### mRNA expression of LOX negatively correlates with miR-141-3p and miR-145-5p expression in renal tissue of patients with ccRCC

**Fig 3:**
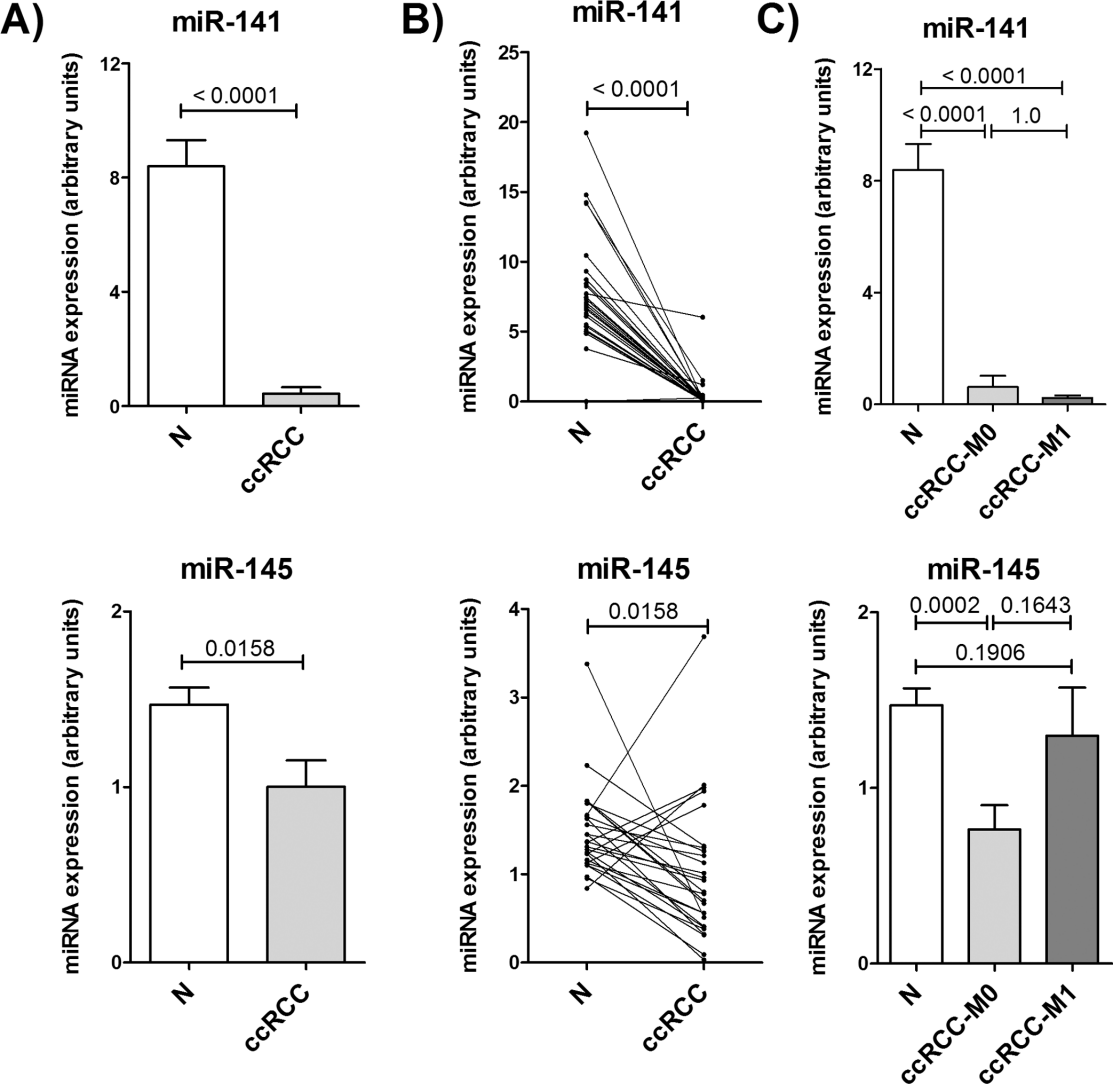
Expression of miR-141-3p and miR-145-5p in ccRCC tissue. Expression of miR-141-3p and miR-145-5p in malignant (ccRCC) and non-malignant (N) renal tissue of 27 ccRCC patients was measured by RT-qPCR. A) and B) show expression differences between paired normal and ccRCC samples. In C) ccRCC tumors are split into non-metastatic (ccRCC-M0; n = 15) and metastatic tumors (ccRCC-M1, n = 12). P-values are given as numbers above bars. Paired samples: Wilcoxon test; unpaired samples: Mann-Whitney test.

As previously described, miR-141-3p and miR-145-5p are substantially lower expressed in ccRCC tissue compared to normal renal tissue [7,39]. We analyzed the miRNA expression in the non-malignant and malignant renal tissue of our patient cohort. Fig 3 shows a decreased expression of both miRNAs. MiR-141-3p was reduced in malignant tissue by 19 fold (Fig 3A). This reduction was observed in all paired tissue samples (Fig 3B), but there was no difference in miR-141-3p expression between ccRCCs with or without metastasis (Fig 3C). The expression of miR-145-5p was 33% lower in ccRCC tissue compared to non-malignant tissue (Fig 3A). Paired tissue showed a reduced expression in tumor tissue for most of the patients, but an increased miR-145-5p expression in 20% (5/27) of the cases (Fig 3B), while four of these five ccRCCs had metastasis.

**Fig 4:**
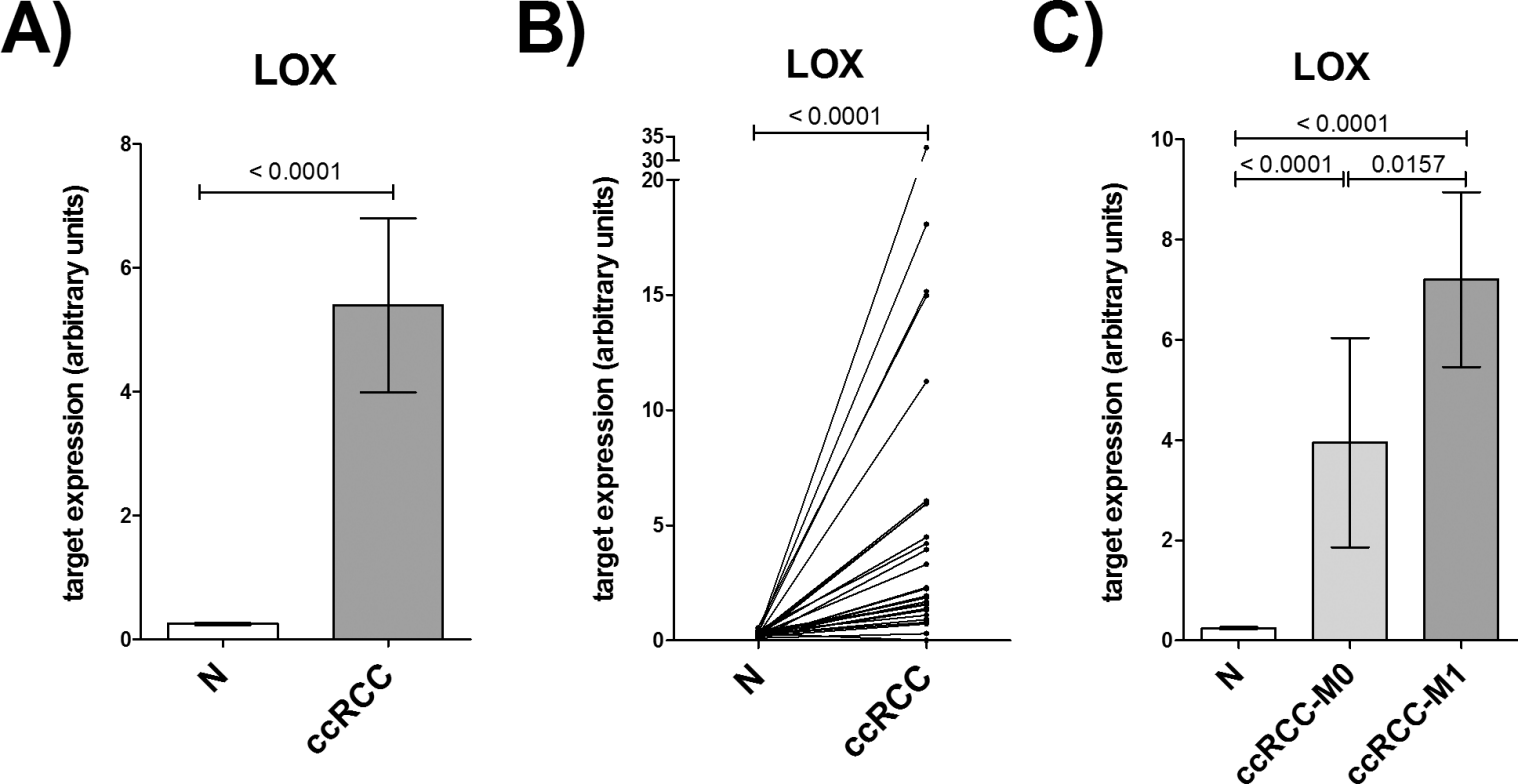
Expression of the target LOX in ccRCC tissue. Expression of LOX in malignant (ccRCC) and non-malignant (N) renal tissue of 27 ccRCC patients was measured by RT-qPCR. A) and B) show expression differences between paired normal and ccRCC samples. In C) ccRCC tumors are split into non-metastatic (ccRCC-M0; n = 15) and metastatic tumors (ccRCC-M1, n = 12). P-values are given as numbers above bars. Paired samples: Wilcoxon test; unpaired samples: Mann-Whitney test.

Because of the reduced expression of miRNAs, we expected an increased target expression in ccRCC tissue. Accordingly, the target LOX showed a significantly enhanced expression in ccRCC vs. non-malignant tissue by 20 fold (Fig 4A). Moreover, LOX expression was significantly higher in tumor tissue of metastatic ccRCCs than in non-metastatic ccRCCs (Fig 4C). These data agree with our microarray expression data revealing a 10 fold higher LOX expression in non-metastatic ccRCC vs. non-malignant tissue and a 16 fold higher LOX expression in metastatic ccRCC vs. non-malignant tissue.

In opposition to our assumption, the other investigated target HS6ST2 showed a major decreased expression in ccRCC tissue in all patients (S3 Fig). HS6ST2 expression in tumor tissue was about 50 fold lower than in normal tissue without any difference between metastatic and non-metastatic tumors. These results could also be confirmed by our microarray data, which also indicated a downregulation of HS6ST2 of almost 50 fold.

By comparing the expression in ccRCC as well as non-malignant tissue, we were able to show a strong correlation between the expression of the target LOX with miRNA expression (S4 Fig). Here the correlation of LOX to both miRNAs miR-141-3p (r_s_ = -0.757****) and miR-145-5p (r_s_ = -0.367**) was negatively directed. This confirms the postulated link between reduced expressed miRNAs and the concomitant increased expression of the corresponding targets.

**Fig 5:**
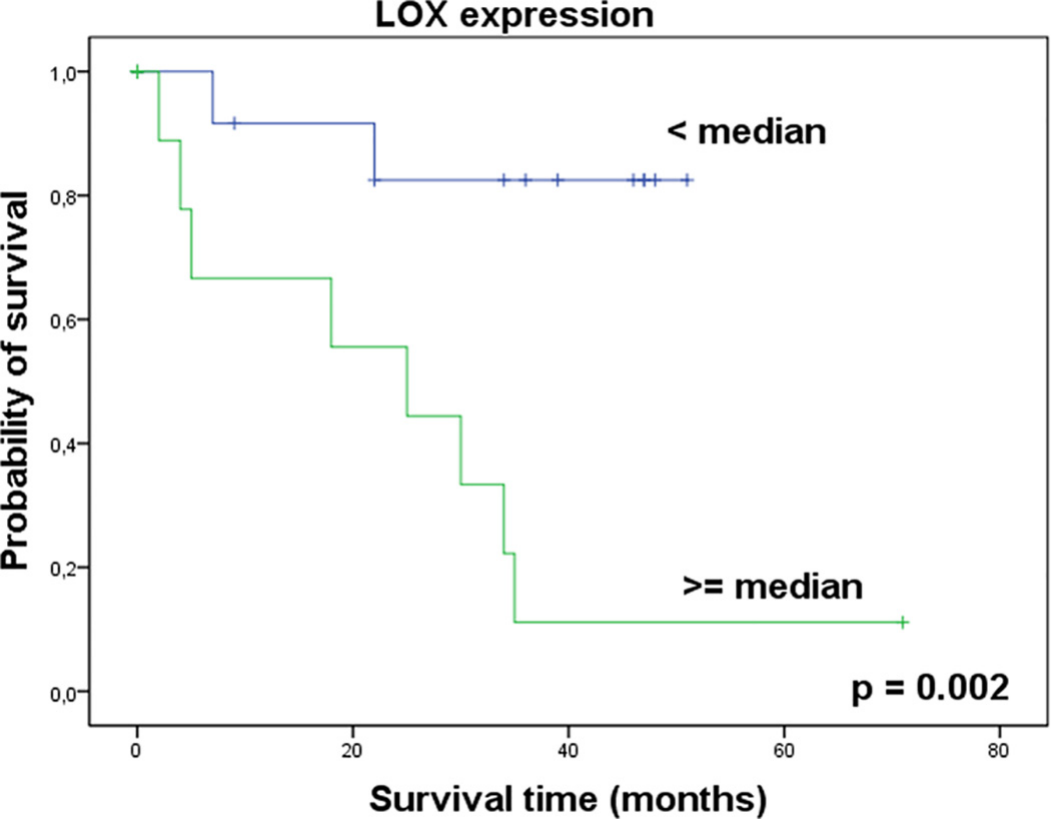
LOX mRNA expression is a prognostic marker in ccRCC. Kaplan-Meier analysis shows LOX mRNA expression in ccRCC tissue connected with overall survival of patients after nephrectomy. Higher LOX expression was significantly associated with a decrease in overall survival. Log rank test.

To test whether the investigated targets LOX and HS6ST2 and miRNAs miR-141-3p and miR-145-5p were able to discriminate between non-malignant and ccRCC tissue or between non-metastatic and metastatic renal tumors, we performed a ROC analysis. As expected from the previous determined expression data, the targets and miRNAs were suitable markers to differentiate between non-malignant and ccRCC tissue (Table 3). However, a differentiation between local ccRCCs and metastatic ccRCCs was only possible using LOX expression. Additionally to these diagnostic features, Kaplan-Meier analysis revealed a strong prognostic significance of LOX mRNA expression in ccRCC tissue for the overall survival of patients (Fig 5).

**Table 3:** ROC analysis to predict the diagnostic impact of the miRNA and mRNA target expression.

Marker	AUC (95% CI)	
	between N and ccRCC	between ccRCC-M0 and ccRCC-M1
HS6ST2	0.974 (0.941–1.000)****	0.697 (0.492–0.902)^ns^
LOX	0.849 (0.725–0.973)****	0.811 (0.644–0.979)**
miR-141	0.912 (0.815–1.000)****	0.539 (0.315–0.763)^ns^
miR-145	0.711 (0.565–0.857)**	0.694 (0.492–0.897)^ns^

AUC = area under the curve; N = non-malignant renal tissue from ccRCC patients; ccRCC = malignant renal tissue from ccRCC patients; ccRCC-M0 = malignant renal tissue from ccRCC patients without metastasis; ccRCC-M1 = malignant renal tissue from ccRCC patients with metastasis

** p < 0.01

**** p < 0.0001; ns = not significant

### Tissue microarray analysis of LOX expression in ccRCC tissue

On mRNA level, LOX expression was significantly increased in ccRCC tissues and correlated with decreased miR-141-3p and miR-145-5p expression. To assess the relevance of LOX protein expression, we evaluated immunostaining on a tissue microarray containing 322 ccRCC tissues. Initially, by analyzing the relation of overall survival after nephrectomy to pathological variables pT and Fuhrman grade (S5 Fig), we could show, that this cohort was representative to analyze the significance of parameters regarding the clinical endpoint ‘overall survival’.

**Fig 6:**
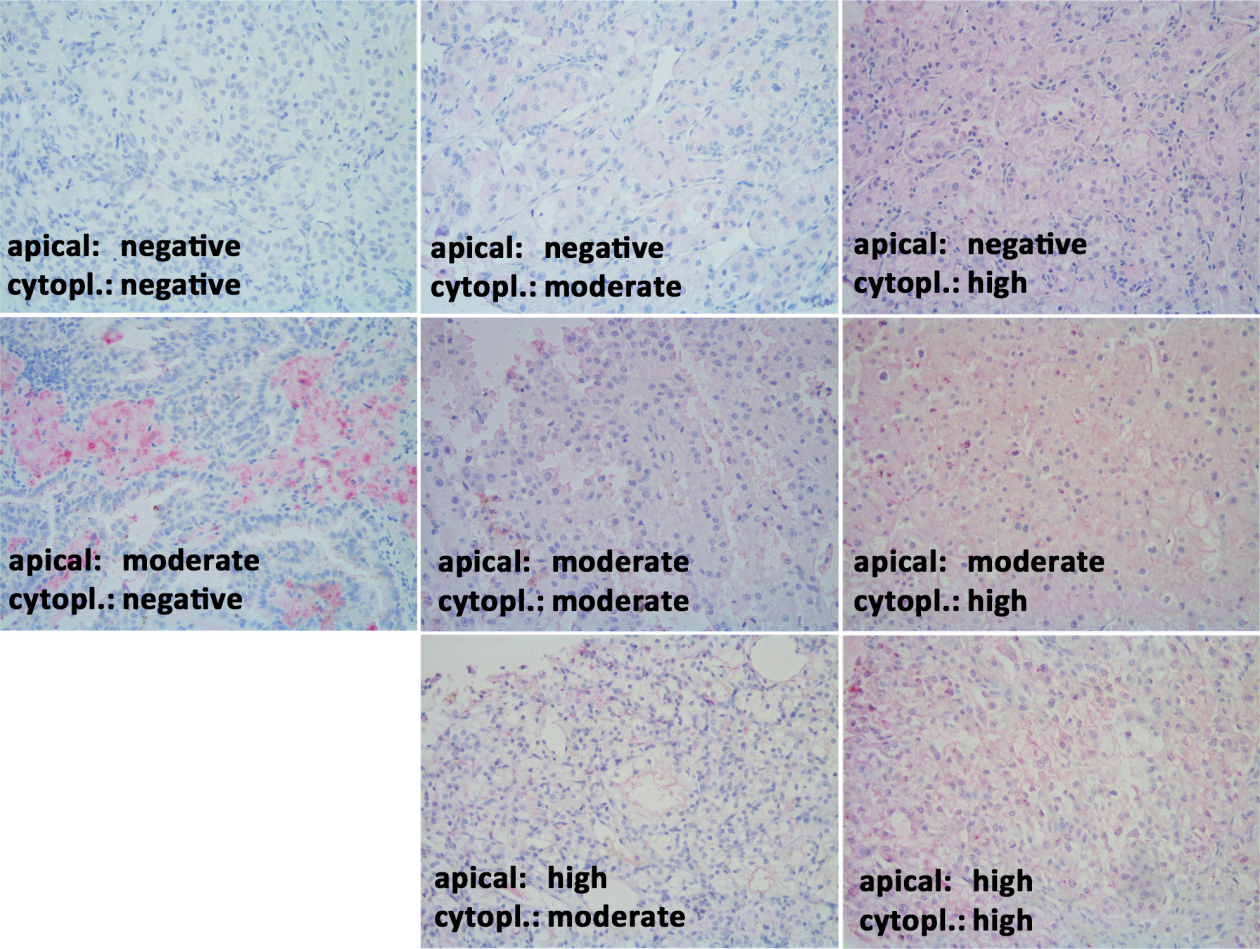
LOX expression in tissue of ccRCC. Apical and cytoplasmic staining were separately scaled in negative, moderately positive or highly positive. There was no tumor with high apical and negative cytoplasmic staining. Magnification: 200x.

**Fig 7:**
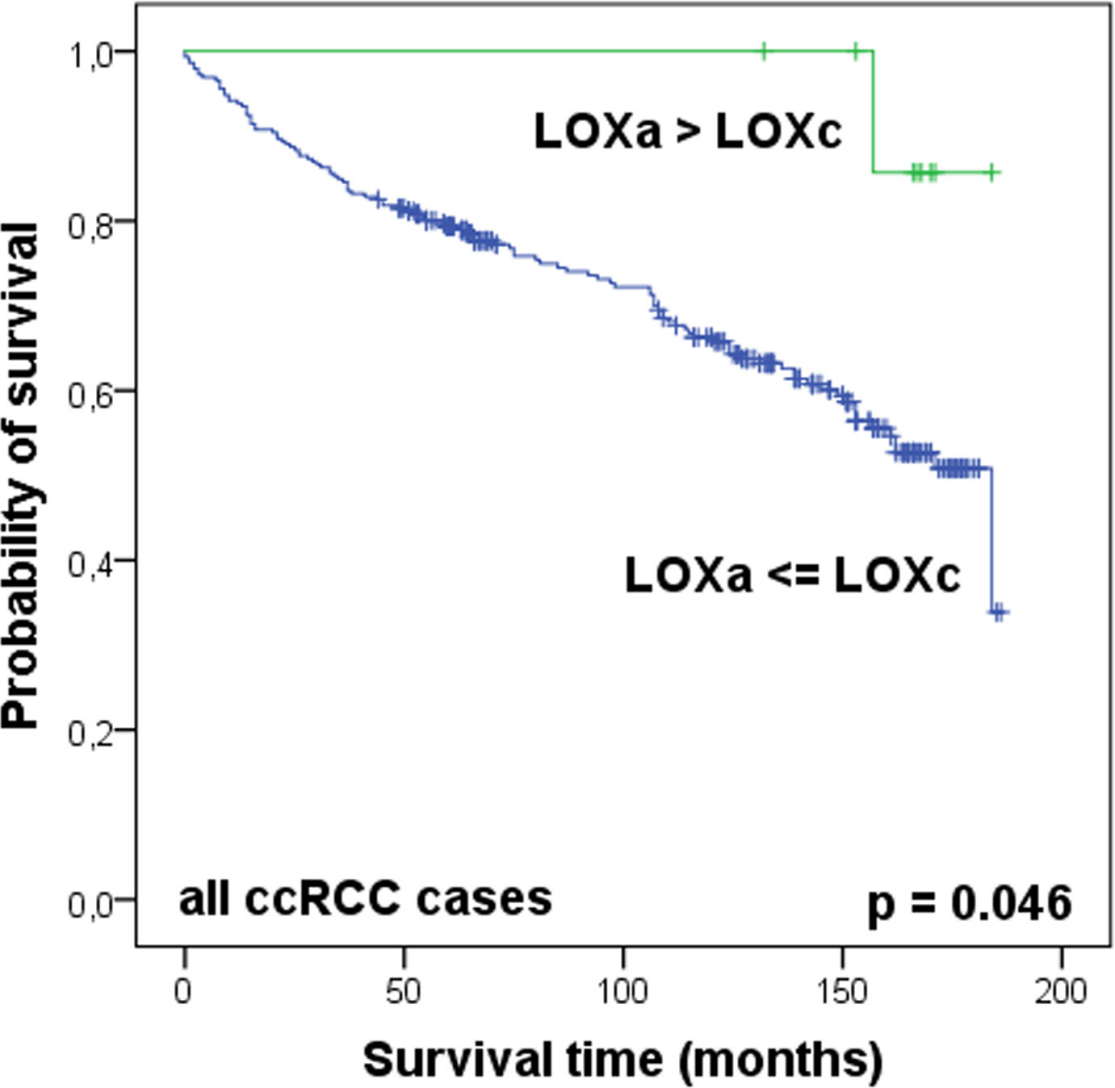
LOX expression pattern seems to be a prognostic marker in ccRCC. Kaplan-Meier analysis shows LOX staining pattern connected with overall survival of the patients after nephrectomy. Higher apical than cytoplasmic LOX staining was significantly associated with an increase in overall survival. Log rank test.

LOX staining was observed in the cytoplasm, on the apical cell membrane and also in intercellular spaces. The assessment of the staining in tumor tissue was performed separately for the apical (LOXa) and cytoplasmic (LOXc) localization with each negative, moderately positive and highly positive staining (Fig 6). LOX staining was evaluable in 301 ccRCCs. In Kaplan-Meier analyses, however, dependence on patient’s survival time could neither be determined for LOXa nor for LOXc (S6 Fig). We also classified tumors in those whose LOXa was greater than LOXc (LOXa > LOXc) and those whose LOXa expression was less than or equal to LOXc (LOXa ≤ LOXc). When comparing the overall survival of patients of these two groups, we observed, that patients with higher LOXa expression (LOXa > LOXc) seemed to have a significant better survival rate than patients with higher or equal LOXc expression (LOXa ≤ LOXc) (p = 0.046) (Fig 7). However, the limiting factor of this classification was the low number of cases in the LOXa > LOXc group with only 9 patients. Clinically, the subdivision of patients with non-metastatic tumors is of particular importance. If metastatic tumors with pN1 and/or M1 were excluded from analyses (all in group LOXa ≤ LOXc), the applied LOX expression level still provided a separation with a tendency to significance (p = 0.071). In the further comparison of LOX expression with clinicopathological data, an association was only found with Fuhrman grade (p = 0.047) (Table 4). Multivariate Cox regression analysis did not find LOX expression as an independent prognostic factor (S5 Table).

**Table 4:** LOX protein expression pattern in ccRCC.

		ccRCC (n = 301)			Chi-square test
		n	LOX_a_ > LOX_z_ (%)	LOX_a_ ≤ LOX_z_ (%)	
ccRCC		301	3	97	
Age	< median	160	3	97	p = 0.884
	> median	141	3	97	
Sex	female	109	1	99	p = 0.112
	male	192	4	96	
pT stage	pT1	180	5	95	p = 0.101
	pT2	14	0	100	
	pT3	105	0	100	
	pT4	2	0	100	
Fuhrman grade	G1	40	10	90	p = 0.047
	G2	222	2	98	
	G3	36	3	97	
	G4	3	0	100	
Nodal status	N0/Nx	283	3	97	p = 0.442
	N1	18	0	100	
Metastases	M0/Mx	272	3	97	p = 0.320
	M1	29	0	100	

## Discussion

Various miRNAs are dysregulated in renal cell carcinoma and are thought to play a central role in tumor formation and progression by altered regulation of their targets. Here we could demonstrate that miR-141-3p and miR-145-5p were downregulated in ccRCC (Fig 3). By identifying the cooperative effect of these miRNAs in the regulation of LOX (Fig 2) and examining their properties as biomarker, we were able to obtain a better insight into the role of miR-141-3p and miR-145-5p and their cooperation in tumor suppressive functions during tumorigenesis of ccRCCs.

Both, miR-141-3p and miR-145-5p are frequently dysregulated in various tumors. While miR-145-5p is solely described to be decreased in tumors, like in cancer of the bladder [40], the prostate [41] and by our previous study and meanwhile by further groups in the kidney [7,8,15], the role of miR-141-3p is less obvious. In tumors of the prostate [41,42], bladder [43], ovary [44] and lung [45] the expression of miR-141-3p is increased and in contrast breast [46,47] gastric [48] tumors show decreased expression. Additionally, in renal tumors miR-141-3p and also the other members of miR-200 family are frequently downregulated [7,11,49–51]. As the reduced expression of miR-141-3p and miR-145-5p is also associated with a shorter tumor-specific [11] or relapse-free survival of ccRCC patients [52], they apparently are involved in pathogenesis of ccRCC although their exact role is still not clear.

It has been described, that both miRNAs have tumor suppressive functions, including the regulation of mechanisms like proliferation, apoptosis, migration and invasion [13–15,53,54]. Also in RCC cell lines including 786-O and ACHN both miRNAs are shown to have an inhibitory effect on cell proliferation [15,55,56]. Additionally, as previously shown [15,53,56] and confirmed by our data, miR-141-3p and miR-145-5p can also inhibit migration and invasion of renal cancer cells (Fig 1). Additionally, we could demonstrate a new, not yet described cooperative function of miR-141-3p and miR-145-5p in the inhibition of cell migration and furthermore a simultaneous and even cooperative regulation of specific oncogenic targets in RCC cell lines (Fig 2). These new targets are already described to be involved in major mechanisms of cancerogenesis like the proliferation promoting targets EAPP and TGFBR2, the anti-apoptotic target VRK2 and the pro-migratory targets HS6ST2 and LOX. So far, there is little information about the expression or functional role of these targets in ccRCC development and progression. But for TGFBR2 it is known that an enhanced activity of the corresponding TGF-β signaling pathway is widely involved in pathogenesis of ccRCC [36]. Microarray data implicate an upregulation of LOX in ccRCC [57,58], which we could confirm in ccRCC tissue by RT-qPCR (Fig 4). Accordingly, the negatively correlated expression of LOX and miR-141-3p and miR-145-5p is in line with our assumption of decreased tumor suppressive miRNAs and the associated increase of oncogenic targets (S4 Fig). As demonstrated here by miR-141-3p and miR-145-5p, the inhibitory outcome of one single miRNA towards target expression can be just minor. However, combining miRNAs with the same target or even with functionally similar targets, a cooperative effect and therefore a more effective inhibition of the appropriate mechanism can be achieved [17] as shown here for the migration of 786-O cells (Fig 1). A cooperative inhibition of a single target by multiple miRNAs was already described [18]. This is possible, if the 3’UTR region of an mRNA transcript comprises the complementary sequence to the seed sequence of different miRNAs (S2 Fig). Hence, for HS6ST2 and LOX we obtained a significant cooperative inhibitory effect in RCC cell lines combining both miRNAs (Fig 2). Such a cooperation of miR-141-3p and miR-145-5p, however, was previously unknown.

In contrast to our expectations, the heparin sulfate sulfotransferase HS6ST2 was reduced expressed in ccRCC tissue (S3 Fig). Thus, the in silico presumed inhibition of HS6ST2 by the miRNAs, later demonstrated in cellular transfection experiments, could not be confirmed in human tissue. But the cooperative inhibitory action of the miRNAs on HS6ST2 might be an important approach in other tumor entities with high HS6ST2 expression like colon [59] or cartilage cancer [60], which however needs to be examined.

The mRNA expression level, as well as the protein localization of the lysyl oxidase LOX indicate a possible prognostic potential in ccRCC tissue (Figs 4–7). LOX belongs to a family of secreted lysyl oxidases, catalyzing inter- and intramolecular crosslink of collagen and elastin in the extracellular matrix [61] and therefore, it plays a central role in the formation and maintenance of extracellular matrix and in the regulation of the structure and cohesion of tissue [62]. Additionally to its function in the extracellular matrix, LOX is thought to carry out tasks in the cytoplasm and even in the nucleus, too, like signal transduction and transcriptional gene regulation [63–68]. The exact role here, however, still needs to be investigated. Recent reports indicate that the cellular localization of LOX protein in tissue of astrocytomas is associated with the malignancy of the tumor [69]. As far as we know, there were no immunohistological analyses of LOX in kidney tissue. Our data suggest, that cellular distribution of LOX protein expression in ccRCC tissue also affects the aggressiveness of the tumor (Fig 7). This could be attributed to the different functions depending on the cell compartment. Albeit limiting for the validity of these results is the small number of ccRCC cases with LOXa > LOXc.

Depending on cell type and transformation status LOX is up- or downregulated in tumors [70–72]. Correspondingly, LOX is described to act as tumor suppressor or promoter of tumor progression. Microarray data implicated an upregulation of LOX in ccRCC [57,58], which we could confirm by RT-qPCR (Fig 4). Using a TMA, however, the findings could not be proven on protein level. Reasons for this inconsistency might be post-transcriptional mechanisms or the in vivo half-life period of the protein [72]. Additionally, LOX is initially secreted into the extracellular matrix and afterwards partly returned into the cell for its intracellular functions [64]. We assessed apical and cytoplasmatic LOX, but secreted LOX in the blood stream or in extracellular lumen cannot be quantitatively detected in the TMA.

LOX seems to be important in a later tumor stage in the transition to metastatic tumors by stiffening the extracellular matrix, reorganization of the cytoskeleton, increasing cell motility [73,74], inducing EMT [75,76], hypoxia-induced migration [32,77] and angiogenesis [78], as well as by setting up pre-metastatic niches [32,79]. Accordingly, LOX is highly expressed mainly in invasive tumors [71] and is probably involved in the transition from localized to metastatic tumors, also in ccRCCs (Fig 4). Therefore, it might represent a promising biomarker for the detection of patients with high risk for metastasis formation or even serve as a target in preventive therapy for these patients, which is already discussed in the literature for tumors in general [80]. But the development of appropriate medications remains still difficult. A possible approach to inhibit LOX expression could be demonstrated in the present study. As described above, we identified LOX as a new target of miR-141-3p and miR-145-5p. A first link between the pro-migratory LOX and miR-145-5p has been described only in cardiovascular diseases [81], but to our knowledge, nothing is known about miR-141-3p or miR-145-5p regulation of LOX in tumors. Additionally, in RCC cells, both miRNAs acted cooperatively on LOX, so that the combination of miR-141-3p and miR-145-5p achieved a drastic inhibition of LOX (Fig 2). Thus, a miRNA replacement therapy, as it is already used in a phase I study by the company Mirna Therapeutics, Inc. with miR-34 [82], would be conceivable and promising.

## Conclusion

Because of the poor prognosis for patients with metastatic RCC, there is still a need for new therapeutic strategies as well as for prognostic and diagnostic markers to identify high risk patients. Here the network of cooperating dysregulated miRNAs and their specific targets provides various promising starting points for new investigations.

The two miRNAs miR-141-3p and miR-145-5p as well as their target LOX have prognostic and diagnostic potential which might lead to new biomarkers. However, especially the newly described cooperative effect of tumor suppressive miRNAs might be a promising approach for the treatment of ccRCC. The combined application of different miRNAs could generate a greater inhibitory effect with fewer side effects. But first of all, an overall understanding of the linked processes is necessary to assess the risks and to develop the best possible strategy. In addition, also the other targets found in our study might be interesting subjects for more extensive investigations.

## Supporting Information

S1 Fig Transient cell transfection with miRNAs. Efficiency of transient cell transfection was verified by different control experiments. A) shows the transfection of 786-O cells with fluorescent labeled miRNA (FAM-labeled Pre-miR Negative Control #1; Life Technologies GmbH, Ambion, Darmstadt, Germany). After transfection FAM-labeled miR was localized in the cytoplasm. B) shows strongly increased miR-145-5p level in 786-O cells 48 h after transfection detected by RT-qPCR. C) To verify the functional activity of transfected miRNA, the direct target of miR-141-3p, ZEB2 and the downstream target CDH1 were detected 48 h after transfection using RT-qPCR. Data are shown as mean (± SEM) relative to NC#1. NC#1 = negative control, n = 3, student’s t-test (two-tailed).
(PDF)

S2 Fig Predicted binding sites on target transcripts. Graphs were generated by TargetScan prediction tool.
LOX:            miR-141-3p (236–242)
                      miR-145-5p (156–162; 827–834)
HS6ST2:      miR-141-3p (155–162; 1802–1808)
                      miR-145-5p (787–793; 1645–1651)
(PDF)

S3 Fig Expression of the target HS6ST2 in ccRCC tissue. Expression of HS6ST2 in malignant (ccRCC) and non-malignant (N) renal tissue of 27 ccRCC patients was measured by RT-qPCR. A) and B) show expression differences between paired normal and ccRCC samples. In C) ccRCC tumors are split into non-metastatic (ccRCC-M0; n = 15) and metastatic tumors (ccRCC-M1, n = 12). P-values are given as numbers above bars. Paired samples: Wilcoxon test; unpaired samples: Mann-Whitney test.
(PDF)

S4 Fig Correlation of expression between miRNAs miR-141-3p and miR-145-5p and targets HS6ST2 and LOX. Correlation between miRNA and target expression in non-malignant and malignant renal tissue of ccRCC patients. r_s_ = Spearman rank correlation coefficients.
(PDF)

S5 Fig Kaplan–Meier analysis of overall survival of ccRCC patients after nephrectomy depending on clinicopathological markers. Log rank test.
(PDF)

S6 Fig Kaplan–Meier analysis of overall survival of ccRCC patients after nephrectomy depending on LOX expression. Log rank test.
(PDF)

S1 Table Assay information for RT-qPCR. (PDF)

S2 Table Assay information for RT-qPCR. (PDF)

S3 Table Synthetic miRNAs. (PDF)

S4 Table Predicted targets for miR-141-3p and miR-145-5p. (PDF)

S5 Table Univariate und multivariate Cox regression for ccRCC patients (pN0M0 only) (n = 284). (PDF)
