# Supplementary material for: Cooperative Effect of miR-141-3p and miR-145-5p in the Regulation of Targets in Clear Cell Renal Cell Carcinoma
Source: PLoS One. 2016 Jun 23;11(6):e0157801. doi: 10.1371/journal.pone.0157801 (PMC4919070; doi:10.1371/journal.pone.0157801)
Supplement: S1 Fig — Efficiency of transient cell transfection was verified by different control experiments. A) shows the transfection of 786-O cells with fluorescent labeled miRNA (FAM-labeled Pre-miR Negative Control #1; Life Technologies GmbH, Ambion, Darmstadt, Germany). After transfection FAM-labeled miR was localized in the cytoplasm. B) shows strongly increased miR-145-5p level in 786-O cells 48 h after transfection detected by RT-qPCR. C) To verify the functional activity of transfected miRNA, the direct target of miR-141-3p, ZEB2 and the downstream target CDH1 were detected 48 h after transfection using RT-qPCR. Data are shown as mean (± SEM) relative to NC#1. NC#1 = negative control, n = 3, student’s t-test (two-tailed). (PDF) [file pone.0157801.s001.pdf]

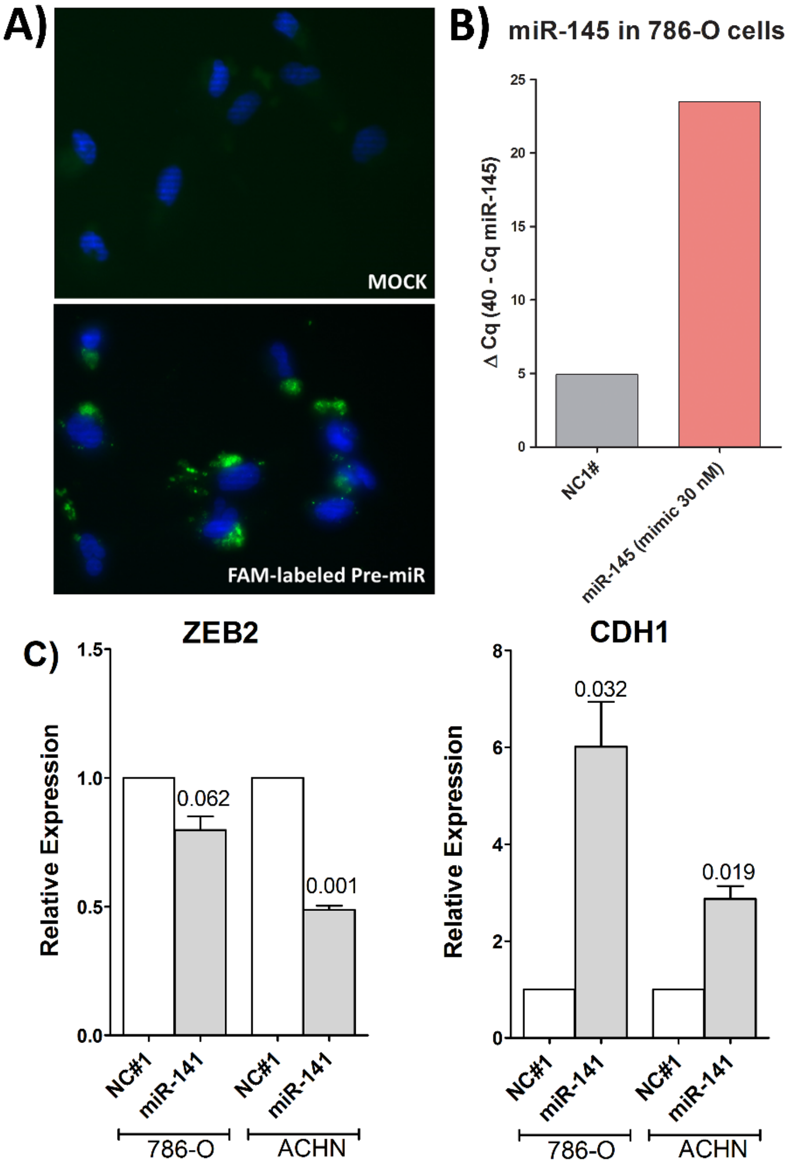


**S1 Fig. Transient cell transfection with miRNAs.** Efficiency of transient cell transfection was verified by different control experiments. A) shows the transfection of 786-O cells with fluorescent labeled miRNA (FAM-labeled Pre-miR Negative Control #1; Life Technologies GmbH, Ambion, Darmstadt, Germany). After transfection FAM-labeled miR was localized in the cytoplasm. B) shows strongly increased miR-145-5p level in 786-O cells 48 h after transfection detected by RT-qPCR. C) To verify the functional activity of transfected miRNA, the direct target of miR-141-3p, ZEB2 and the downstream target CDH1 were detected 48 h after transfection using RT-qPCR. Data are shown as mean (± SEM) relative to NC#1. NC#1 = negative control, n = 3, student's t-test (two-tailed).
