# Supplementary material for: Cooperative Effect of miR-141-3p and miR-145-5p in the Regulation of Targets in Clear Cell Renal Cell Carcinoma
Source: PLoS One. 2016 Jun 23;11(6):e0157801. doi: 10.1371/journal.pone.0157801 (PMC4919070; doi:10.1371/journal.pone.0157801)
Supplement: S3 Fig — Expression of HS6ST2 in malignant (ccRCC) and non-malignant (N) renal tissue of 27 ccRCC patients was measured by RT-qPCR. A) and B) show expression differences between paired normal and ccRCC samples. In C) ccRCC tumors are split into non-metastatic (ccRCC-M0; n = 15) and metastatic tumors (ccRCC-M1, n = 12). P-values are given as numbers above bars. Paired samples: Wilcoxon test; unpaired samples: Mann-Whitney test. (PDF) [file pone.0157801.s003.pdf]

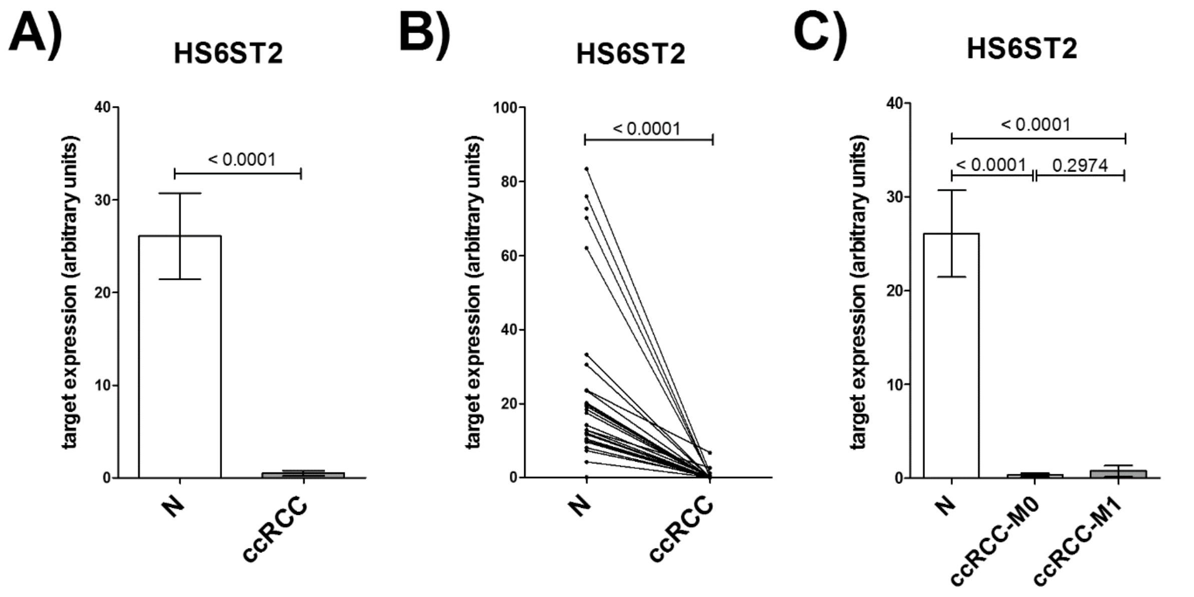

S3 Fig. Expression of the target HS6ST2 in ccRCC tissue. Expression of HS6ST2 in malignant (ccRCC) and non-malignant (N) renal tissue of 27 ccRCC patients was measured by RT-qPCR. A) and B) show expression differences between paired normal and ccRCC samples. In C) ccRCC tumors are split into non-metastatic (ccRCC-M0; n = 15) and metastatic tumors (ccRCC-M1, n = 12). P-values are given as numbers above bars. Paired samples: Wilcoxon test; unpaired samples: Mann-Whitney test.
