# Supplementary material for: Cooperative Effect of miR-141-3p and miR-145-5p in the Regulation of Targets in Clear Cell Renal Cell Carcinoma
Source: PLoS One. 2016 Jun 23;11(6):e0157801. doi: 10.1371/journal.pone.0157801 (PMC4919070; doi:10.1371/journal.pone.0157801)
Supplement: S4 Fig — Correlation between miRNA and target expression in non-malignant and malignant renal tissue of ccRCC patients. rs = Spearman rank correlation coefficients. (PDF) [file pone.0157801.s004.pdf]

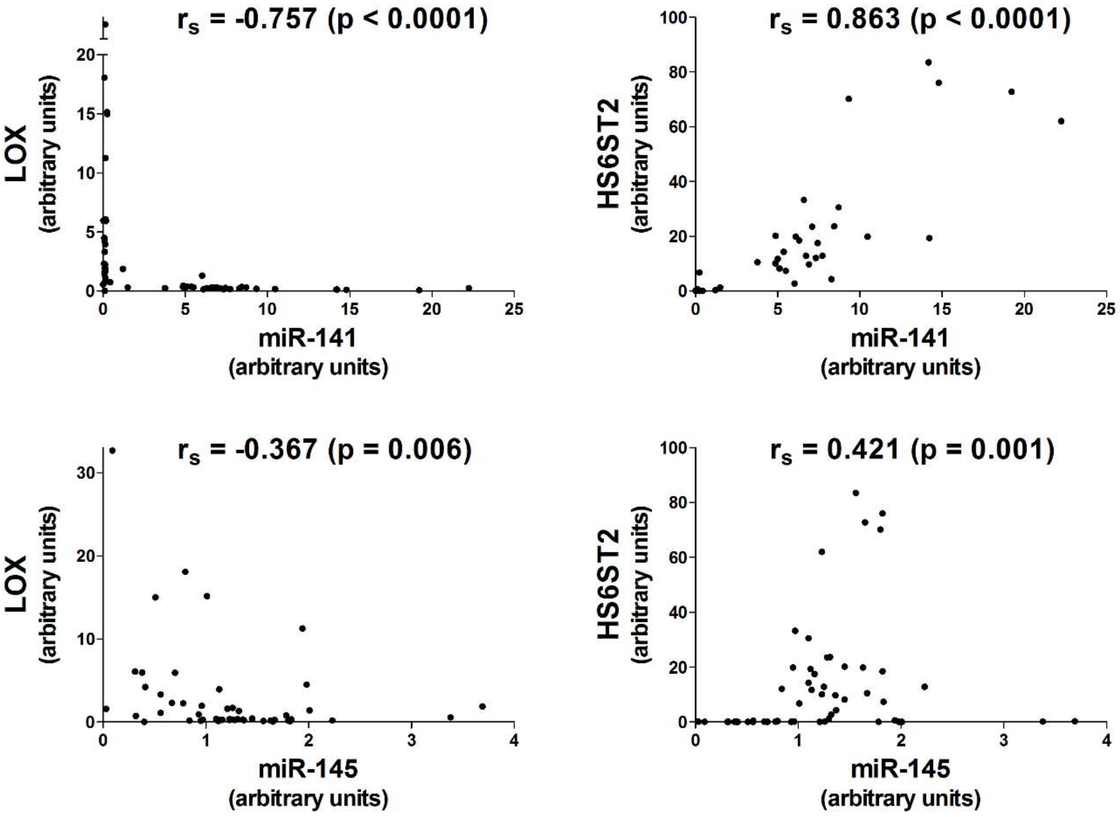

S4 Fig. Correlation of expression between miRNAs miR-141-3p and miR-145-5p and targets HS6ST2 and LOX. Correlation between miRNA and target expression in non-malignant and malignant renal tissue of ccRCC patients. r_s = Spearman rank correlation coefficients.
