# Supplementary material for: Cooperative Effect of miR-141-3p and miR-145-5p in the Regulation of Targets in Clear Cell Renal Cell Carcinoma
Source: PLoS One. 2016 Jun 23;11(6):e0157801. doi: 10.1371/journal.pone.0157801 (PMC4919070; doi:10.1371/journal.pone.0157801)
Supplement: S5 Fig — Log rank test. (PDF) [file pone.0157801.s005.pdf]

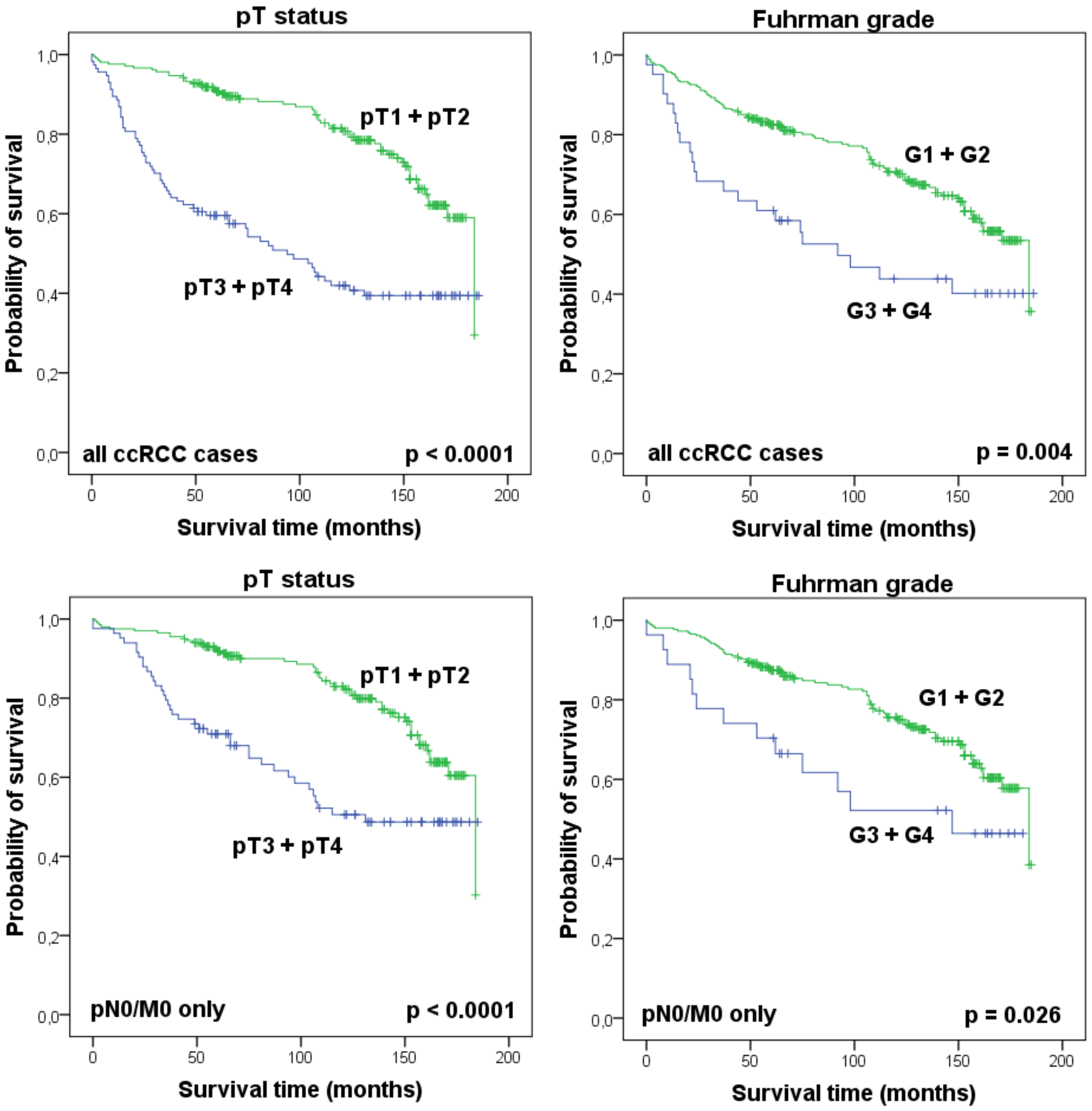

S5 Fig. Kaplan–Meier analysis of overall survival of ccRCC patients after nephrectomy depending on clinicopathological markers. Log rank test.
