# Supplementary material for: Cooperative Effect of miR-141-3p and miR-145-5p in the Regulation of Targets in Clear Cell Renal Cell Carcinoma
Source: PLoS One. 2016 Jun 23;11(6):e0157801. doi: 10.1371/journal.pone.0157801 (PMC4919070; doi:10.1371/journal.pone.0157801)
Supplement: S1 Table — (PDF) [file pone.0157801.s007.pdf]

S1 Table. Assay information for RT-qPCR.

Gene	Forward (5´-3´)	Reverse (5´-3´)	Assay	Tm
EAPP	TTCCGGATGACTACGACCCC	AGCACATCCACTTCATCCTCAG	SYBR	57°C
FRMD4A	AGCAAGGGGAAGATCATCAG	CGACTGCGAGCTATCTGATTC	UPL #40	60°C
HS6ST2	TGCGATCTTCTCCAAGATTTTC	CGATCACGGCAAATAGGAAG	UPL #9	60 °C
ITGB3	AGCTCATTGTTGATGCTTATGG	AACTCTTCAGGGAGGTCACG	UPL #11	60°C
LOX	TGGCCGACCCCTACTACATC	ACATCTGCCCTGTATGCTGT	SYBR	59 °C
NRP2	GGTATACCCGGAGAGGTGGT	TCTACCGTGGGCTTGGAGT	UPL #26	60°C
SLC16A3	GAGTTTGGGATCGGCTACAG	CGGTTCACGCACACACTG	UPL #58	60°C
TGFB2	ATAGACATGCCGCCCTTCTT	GGCATCAAGGTACCCACAGA	SYBR	57°C
TNFSF4	GCTCCTGTGCTTCACCTACA	CCTCCTTTTGGGAAGTGAGGAT	SYBR	57°C
VRK2	AAGTGGATAGAACGCAAACAACT	CCTAGTCTTTCCATTACCATAAATCTG	UPL #6	60°C
PPIA	Hs_PPIA_1_SG QuantiTect Primer Assay			55°C
