# Supplementary material for: Cooperative Effect of miR-141-3p and miR-145-5p in the Regulation of Targets in Clear Cell Renal Cell Carcinoma
Source: PLoS One. 2016 Jun 23;11(6):e0157801. doi: 10.1371/journal.pone.0157801 (PMC4919070; doi:10.1371/journal.pone.0157801)
Supplement: S2 Table — (PDF) [file pone.0157801.s008.pdf]

S2 Table. Assay information for RT-qPCR.

Name	Name miRBase 21	Accession # miRBase 21	Mature Sequence	Assay ID
miR-28	hsa-miR-28-5p	MIMAT0000085	AAGGAGCUCACAGUCUAUUGAG	000 411
miR-103	hsa-miR-103a-3p	MIMAT0000101	AGCAGCAUUGUACAGGGCUAUGA	000 439
miR-106a	hsa-miR-106a-5p	MIMAT0000103	AAAAGUGCUUACAGUGCAGGUAG	00 2169
miR-141	hsa-miR-141-3p	MIMAT0000432	UAACACUGUCUGGUAAAGAUGG	000463
miR-145	hsa-miR-145-5p	MIMAT0000437	GUCCAGUUUUCCCAGGAAUCCCU	00 2278
