# Supplementary material for: Cooperative Effect of miR-141-3p and miR-145-5p in the Regulation of Targets in Clear Cell Renal Cell Carcinoma
Source: PLoS One. 2016 Jun 23;11(6):e0157801. doi: 10.1371/journal.pone.0157801 (PMC4919070; doi:10.1371/journal.pone.0157801)
Supplement: S3 Table — (PDF) [file pone.0157801.s009.pdf]

S3 Table. Synthetic miRNAs.

miRNA	Assay	Assay ID
miR-141-3p	miRVana™ miRNA mimic	MC10860
miR-145-5p	miRVana™ miRNA mimic	MC11480
NC#1	mirVana™ miRNA Mimic, Negative Control #1	4464058
