# Supplementary material for: Cooperative Effect of miR-141-3p and miR-145-5p in the Regulation of Targets in Clear Cell Renal Cell Carcinoma
Source: PLoS One. 2016 Jun 23;11(6):e0157801. doi: 10.1371/journal.pone.0157801 (PMC4919070; doi:10.1371/journal.pone.0157801)
Supplement: S4 Table — (PDF) [file pone.0157801.s010.pdf]

S4 Table. Predicted targets for miR-141-3p and miR-145-5p.

Gene	RefseqID	Gene Name
ACP2	NM_001610	ACP2 acid phosphatase 2, lysosomal [Homo sapiens (human)]
ADAMT	NM_007037	ADAMTS8 ADAM metallopeptidase with thrombospondin type 1 motif, 8 [Homo
AHR	NM_001621	AHR aryl hydrocarbon receptor [Homo sapiens (human)]
AK2	NM_013411	AK2 adenylate kinase 2 [Homo sapiens (human)]
ALG2	NM_033087	ALG2 ALG2, alpha-1,3/1,6-mannosyltransferase [Homo sapiens (human)]
ALS2	NM_020919	ALS2 amyotrophic lateral sclerosis 2 (juvenile) [Homo sapiens (human)]
APH1B	NM_031301	APH1B APH1B gamma secretase subunit [Homo sapiens (human)]
ARMC7	NM_024585	ARMC7 armadillo repeat containing 7 [Homo sapiens (human)]
ATXN1	NM_000332	ATXN1 ataxin 1 [Homo sapiens (human)]
ATXN7L	NM_020725	ATXN7L1 ataxin 7-like 1 [Homo sapiens (human)]
C10orf1	NM_018017	C10orf118 chromosome 10 open reading frame 118 [Homo sapiens (human)]
C10orf4	NM_153256	PROSER2 proline and serine rich 2 [Homo sapiens (human)]
C14orf4	NM_152592	SYNE3 spectrin repeat containing, nuclear envelope family member 3 [Homo
C1QTNF	NM_015645	C1QTNF5 C1q and tumor necrosis factor related protein 5 [Homo sapiens
C6orf11	NM_033069	GFOD1 glucose-fructose oxidoreductase domain containing 1 [Homo sapiens
C9orf12	NM_173690	SCAI suppressor of cancer cell invasion [Homo sapiens (human)]
C9orf16	NM_198573	ENHO energy homeostasis associated [Homo sapiens (human)]
CCDC28	NM_015439	CCDC28A coiled-coil domain containing 28A [Homo sapiens (human)]
CGNL1	NM_032866	CGNL1 cingulin-like 1 [Homo sapiens (human)]
CIAO1	NM_004804	CIAO1 cytosolic iron-sulfur assembly component 1 [Homo sapiens (human)]
DDHD1	NM_030637	DDHD1 DDHD domain containing 1 [Homo sapiens (human)]
DNHD1	NM_144666	DNHD1 dynein heavy chain domain 1 [Homo sapiens (human)]
DOK6	NM_152721	DOK6 docking protein 6 [Homo sapiens (human)]
DPH3	NM_001047434	DPH3 diphthamide biosynthesis 3 [Homo sapiens (human)]
DUSP6	NM_001946	DUSP6 dual specificity phosphatase 6 [Homo sapiens (human)]
EAPP	NM_018453	EAPP E2F-associated phosphoprotein [Homo sapiens (human)]
ELOVL2	NM_017770	ELOVL2 ELOVL fatty acid elongase 2 [Homo sapiens (human)]
EPN1	NM_001130071	EPN1 epsin 1 [Homo sapiens (human)]
FRMD4A	NM_018027	FRMD4A FERM domain containing 4A [Homo sapiens (human)]
FUS	NM_004960	FUS FUS RNA binding protein [Homo sapiens (human)]
GABAR	NM_007285	GABARAPL2 GABA(A) receptor-associated protein-like 2 [Homo sapiens
GLG1	NM_012201	GLG1 golgi glycoprotein 1 [Homo sapiens (human)]
GLULD1	NM_016571	LGSN lengsin, lens protein with glutamine synthetase domain [Homo sapiens
GOLM1	NM_016548	GOLM1 golgi membrane protein 1 [Homo sapiens (human)]
GPR137	NM_001099652	GPR137C G protein-coupled receptor 137C [Homo sapiens (human)]
GRB10	NM_001001555	GRB10 growth factor receptor-bound protein 10 [Homo sapiens (human)]
GTF2H5	NM_207118	GTF2H5 general transcription factor IIH, polypeptide 5 [Homo sapiens (human)]
HDAC8	NM_018486	HDAC8 histone deacetylase 8 [Homo sapiens (human)]
HS6ST2	NM_001077188	HS6ST2 heparan sulfate 6-O-sulfotransferase 2 [Homo sapiens (human)]
HTATSF	NM_014500	HTATSF1 HIV-1 Tat specific factor 1 [Homo sapiens (human)]
HTR2C	NM_000868	HTR2C 5-hydroxytryptamine (serotonin) receptor 2C, G protein-coupled [Homo
IRS2	NM_003749	IRS2 insulin receptor substrate 2 [Homo sapiens (human)]
ITGB3	NM_000212	ITGB3 integrin, beta 3 (platelet glycoprotein IIIa, antigen CD61) [Homo sapiens
KIAA160	NM_020940	FAM160B1 family with sequence similarity 160, member B1 [Homo sapiens
KLF5	NM_001730	KLF5 Kruppel-like factor 5 (intestinal) [Homo sapiens (human)]
LMLN	NM_033029	LMLN leishmanolysin-like (metallopeptidase M8 family) [Homo sapiens (human)]
LOC440	NM_001013698	SMCO3 single-pass membrane protein with coiled-coil domains 3 [Homo sapiens
LOX	NM_002317	LOX lysyl oxidase [Homo sapiens (human)]
LZIC	NM_032368	LZIC leucine zipper and CTNNBIP1 domain containing [Homo sapiens (human)]
MDFIC	NM_199072	MDFIC MyoD family inhibitor domain containing [Homo sapiens (human)]
MEGF11	NM_032445	MEGF11 multiple EGF-like-domains 11 [Homo sapiens (human)]
MGC338	NM_175885	FAM181B family with sequence similarity 181, member B [Homo sapiens
MKL2	NM_014048	MKL2 MKL/myocardin-like 2 [Homo sapiens (human)]
MON2	NM_015026	MON2 MON2 homolog (S. cerevisiae) [Homo sapiens (human)]
MTMR15	NM_014967	FAN1 FANCD2/FANCI-associated nuclease 1 [Homo sapiens (human)]
MX1	NM_002462	MX1 myxovirus (influenza virus) resistance 1, interferon-inducible protein p78
NEK11	NM_024800	NEK11 NIMA-related kinase 11 [Homo sapiens (human)]
NPC1	NM_000271	NPC1 Niemann-Pick disease, type C1 [Homo sapiens (human)]
NRIP3	NM_020645	NRIP3 nuclear receptor interacting protein 3 [Homo sapiens (human)]
NRP2	NM_201266	NRP2 neuropilin 2 [Homo sapiens (human)]

**OSBPL9**	NM_148909	OSBPL9 oxysterol binding protein-like 9 [Homo sapiens (human)]
**PAN2**	NM_001127460	PAN2 PAN2 poly(A) specific ribonuclease subunit [Homo sapiens (human)]
**PAPD4**	NM_001114394	PAPD4 PAP associated domain containing 4 [Homo sapiens (human)]
**PAQR9**	NM_198504	PAQR9 progestin and adipoQ receptor family member IX [Homo sapiens
**PDCD4**	NM_145341	PDCD4 programmed cell death 4 (neoplastic transformation inhibitor) [Homo
**PLAG1**	NM_002655	PLAG1 pleiomorphic adenoma gene 1 [Homo sapiens (human)]
**PNPLA6**	NM_006702	PNPLA6 patatin-like phospholipase domain containing 6 [Homo sapiens (human)
**PPM1L**	NM_139245	PPM1L protein phosphatase, Mg2+/Mn2+ dependent, 1L [Homo sapiens
**PRTG**	NM_173814	PRTG protogenin [Homo sapiens (human)]
**PTGDR**	NM_000953	PTGDR prostaglandin D2 receptor (DP) [Homo sapiens (human)]
**RASAL2**	NM_170692	RASAL2 RAS protein activator like 2 [Homo sapiens (human)]
**RBPMS2**	NM_194272	RBPMS2 RNA binding protein with multiple splicing 2 [Homo sapiens (human)]
**RIMS1**	NM_014989	RIMS1 regulating synaptic membrane exocytosis 1 [Homo sapiens (human)]
**RPA2**	NM_002946	RPA2 replication protein A2, 32kDa [Homo sapiens (human)]
**SCARB2**	NM_005506	SCARB2 scavenger receptor class B, member 2 [Homo sapiens (human)]
**SCML4**	NM_198081	SCML4 sex comb on midleg-like 4 (Drosophila) [Homo sapiens (human)]
**SLC16A**	NM_001042422	SLC16A3 solute carrier family 16 (monocarboxylate transporter), member 3 [
**SLC25A**	NM_031291	SLC25A31 solute carrier family 25 (mitochondrial carrier; adenine nucleotide
**SNED1**	NM_001080437	SNED1 sushi, nidogen and EGF-like domains 1 [Homo sapiens (human)]
**SPG3A**	NM_001127713	ATL1 atlastin GTPase 1 [Homo sapiens (human)]
**SRP9**	NM_001130440	SRP9 signal recognition particle 9kDa [Homo sapiens (human)]
**SYPL2**	NM_001040709	SYPL2 synaptophysin-like 2 [Homo sapiens (human)]
**TARSL2**	NM_152334	TARSL2 threonyl-tRNA synthetase-like 2 [Homo sapiens (human)]
**TBC1D2**	NM_144572	TBC1D2B TBC1 domain family, member 2B [Homo sapiens (human)]
**TGFB2**	NM_003238	TGFB2 transforming growth factor, beta 2 [Homo sapiens (human)]
**THEM2**	NM_018473	ACOT13 acyl-CoA thioesterase 13 [Homo sapiens (human)]
**TMEM16**	NM_001025356	ANO6 anoctamin 6 [Homo sapiens (human)]
**TMEM42**	NM_144638	TMEM42 transmembrane protein 42 [Homo sapiens (human)]
**TNFSF4**	NM_003326	TNFSF4 tumor necrosis factor (ligand) superfamily, member 4 [Homo sapiens
**TRIM65**	NM_173547	TRIM65 tripartite motif containing 65 [Homo sapiens (human)]
**TTC14**	NM_001042601	TTC14 tetratricopeptide repeat domain 14 [Homo sapiens (human)]
**USP13**	NM_003940	USP13 ubiquitin specific peptidase 13 (isopeptidase T-3) [Homo sapiens
**VRK2**	NM_001130483	VRK2 vaccinia related kinase 2 [Homo sapiens (human)]
**WDFY3**	NM_014991	WDFY3 WD repeat and FYVE domain containing 3 [Homo sapiens (human)]
**WDR37**	NM_014023	WDR37 WD repeat domain 37 [Homo sapiens (human)]
**WDR43**	NM_015131	WDR43 WD repeat domain 43 [Homo sapiens (human)]
**YTHDF2**	NM_016258	YTHDF2 YTH domain family, member 2 [Homo sapiens (human)]
**ZNF24**	NM_006965	ZNF24 zinc finger protein 24 [Homo sapiens (human)]
**ZNF398**	NM_020781	ZNF398 zinc finger protein 398 [Homo sapiens (human)]
**ZNF830**	NM_052857	ZNF830 zinc finger protein 830 [Homo sapiens (human)]

Selected targets for further analysis are shaded in gray.
