# Supplementary material for: Cooperative Effect of miR-141-3p and miR-145-5p in the Regulation of Targets in Clear Cell Renal Cell Carcinoma
Source: PLoS One. 2016 Jun 23;11(6):e0157801. doi: 10.1371/journal.pone.0157801 (PMC4919070; doi:10.1371/journal.pone.0157801)
Supplement: S5 Table — (PDF) [file pone.0157801.s011.pdf]

S5 Table. Univariate und multivariate Cox regression for ccRCC patients (pN0M0 only) (n = 284).

	Criteria	univariate analysis		multivariate analysis (inclusion)[a]			
				only clinicopathological variables		clinicopathological variables and targets	
		HR	p-value	HR	p-value	HR	p-value
Age	continuous	1.053	< 0.0001	1.052	< 0.0001	1.053	< 0.0001
Sex	female<br>male	0.747	0.202				
pT status	pT1-2<br>pT3-4	2.398	< 0.0001	2.160	0.001	1.956	0.004
Fuhrman grade	G1-2<br>G3-4	1.934	0.029	1.527	0.171	1.596	0.135
LOX	$LOX_a > LOX_z$<br>$LOX_a \leq LOX_z$	0.195	0.105			0.242	0.162

HR = Hazard Ratio

[a] only variables which were significant in the univariate analysis are included in multivariate models
