# Supplementary material for: Cooperative Effect of miR-141-3p and miR-145-5p in the Regulation of Targets in Clear Cell Renal Cell Carcinoma
Source: PLoS One. 2016 Jun 23;11(6):e0157801. doi: 10.1371/journal.pone.0157801 (PMC4919070; doi:10.1371/journal.pone.0157801)
Supplement: S2 Fig — Graphs were generated by TargetScan prediction tool. LOX: miR-141-3p (236–242) miR-145-5p (156–162; 827–834) HS6ST2: miR-141-3p (155–162; 1802–1808) miR-145-5p (787–793; 1645–1651) (PDF) [file pone.0157801.s002.pdf]

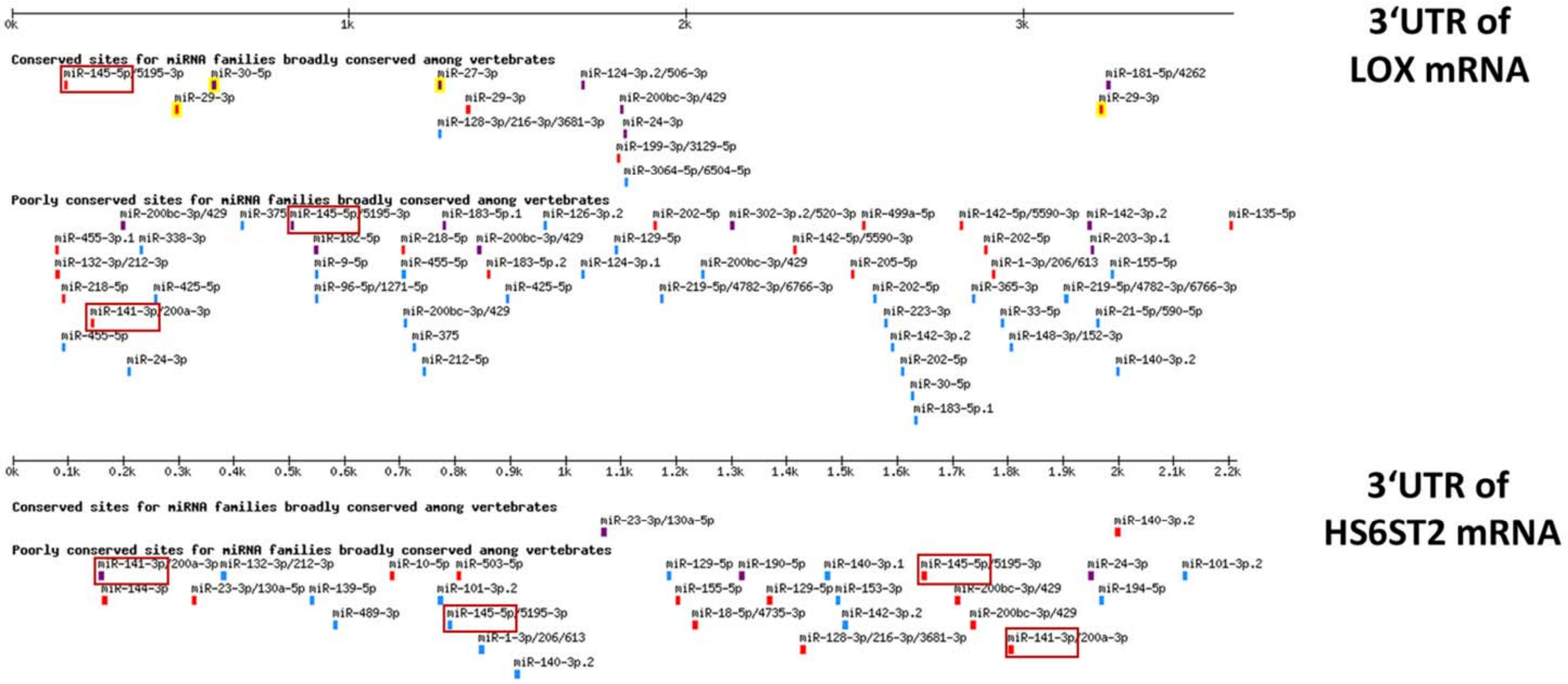

S2 Fig. Predicted binding sites on target transcripts.

Graphs were generated by TargetScan prediction tool.

LOX: miR-141-3p (236-242)

miR-145-5p (156-162; 827-834)

HS6ST2: miR-141-3p (155-162; 1802-1808)

miR-145-5p (787-793; 1645-1651)
